# Perturbations in the Replication Program Contribute to Genomic Instability in Cancer

**DOI:** 10.3390/ijms18061138

**Published:** 2017-05-25

**Authors:** Britny Blumenfeld, Micha Ben-Zimra, Itamar Simon

**Affiliations:** 1 Department of Microbiology and Molecular Genetics, IMRIC, Faculty of Medicine, Hebrew University of Jerusalem, Jerusalem 91120, Israel; britny.blumenfeld@mail.huji.ac.il (B.B.); micha.ben-zimra@mail.huji.ac.il (M.B.-Z.); 2 Pharmacology and Experimental Therapeutics Unit, The Institute for Drug Research, School of Pharmacy, Faculty of Medicine, The Hebrew University of Jerusalem, Jerusalem 91120, Israel

**Keywords:** genomic instability, replication timing, replication stress, cancer

## Abstract

Cancer and genomic instability are highly impacted by the deoxyribonucleic acid (DNA) replication program. Inaccuracies in DNA replication lead to the increased acquisition of mutations and structural variations. These inaccuracies mainly stem from loss of DNA fidelity due to replication stress or due to aberrations in the temporal organization of the replication process. Here we review the mechanisms and impact of these major sources of error to the replication program.

## 1. Introduction

Malignant transformation is characterized by uncontrolled cell growth, which is often caused by an accumulation of mutations due to increased mutation rates and genome instability. Accurate deoxyribonucleic acid (DNA) replication is crucial for preventing such genomic instability [1,2] since during replication there is great potential for the introduction of mutations. Three main mechanisms contribute to mutations during DNA replication: (1) Mismatches inserted during replication due to the inherent error rates in DNA polymerase [3] that escaped mismatch repair [4]; (2) DNA damage that occurred prior to replication initiation that was not repaired in time; and (3) DNA damage that occurred during replication itself [5].

In this review we will discuss two major types of changes in replication that may lead to genome instability. The first potential alteration of the replication program is an increase in replication stress, which is defined as replication fork slowing, stalling, or collapse. Heightened replication stress frequently leads to higher levels of DNA damage and genomic instability [6]. This phenomenon plays an important role in the development of cancer as proposed by the oncogene-induced replication stress model. According to this model, cancerous transformation is driven by increased replication stress, which creates an environment of genomic instability [7]. This model suggests that the increased mutation rate essential for cancer development stems from replication stress rather than from random mutations in genes involved in genome surveillance [8]. The oncogene-induced replication stress model is supported by recent high-throughput sequencing studies that discovered a paucity of mutations in DNA repair genes in primary cancer [7].

The second replication-related change linked to genomic instability involves changes in the temporal organization of the replication process, also known as replication timing. DNA replication is a strictly regulated process, in which origins of replication fire at distinct times during synthesis (S) phase creating distinct early and late replication zones [9]. Aberrant order of replication initiation has been implicated in cancer and in the generation of genomic instability [10,11], though the frequency and significance of such changes during cancer transformation is not yet clear.

The relationship between replication stress and replication timing is highly complex and interdependent. Nonetheless, replication stress and replication timing remain distinct. Replication stress refers to the fidelity of the replication fork, whereas replication timing refers to the relative time at which different regions are replicated. Changes in replication timing may potentially result in discoordination of the replication process and lead to stress. Conversely, replication stress may affect the replication timing program as many stalled or collapsed forks in a given region may delay the time at which it is replicated. However, in yeast it was shown that induction of replication stress through depletion of the nucleotide pool or through mutations in certain cell cycle proteins, can alter the duration of the replication but does not affect the general replication timing profile [12,13]. Experiments validating this in mammals remain to be performed.

## 2. Replication Stress

### 2.1. Mechanisms of Replication Stress

During S phase of the cell cycle, the cell must accurately replicate its entire DNA content. Eukaryotic replication is initiated from multiple licensed origins and expands via bidirectional replication forks in order to fully replicate the entire genome [14]. Replication stress occurring during this process results in slow or stalled DNA replication forks [15] and can prevent accurate replication of DNA. These stalled or collapsed forks expose single stranded DNA (ssDNA) as the helicase continues to unwind the adjacent DNA, leaving the region susceptible to double strand breaks (DSB) [6,16,17]. Normally, to prevent such damage, replication protein A (RPA) coats the ssDNA and activates ataxia telangiectasia and Rad3-related protein (ATR) initiating a cascade of events to resolve the stalled forks or bypass collapsed forks [18]. Failure in this DNA repair pathway will lead to cell cycle arrest, whereas partial recovery from stalled forks may lead to genomic instability. Thus, high levels of replication stress frequently result in the accumulation of mutations as well as larger chromosomal aberrations such as copy number variations and translocations [6,19].

There are a number of potential sources of replication stress (reviewed in [18,20]). First, replication stress can be caused by direct barriers to the replication fork. This includes lesions in the DNA induced by UV light or chemical mutagens [21], which physically block replication fork progression. Production of reactive oxygen species (ROS) can result in oxidized or abasic sites, which will impede fork progression [22]. Repetitive regions or unusual DNA structures such as the G-quadruplex are also challenging to fork progression [23]. Additionally, accidental incorporation of ribonucleotides in the DNA can serve as a barrier to fork progression [24].

Another major source of replication stress is a lack of resources. The DNA replication program requires many different factors and fork progression can be limited by their deficiency. Adequate levels of deoxynucleotide triphosphates (dNTPs) are crucial for the progression of the replication fork [25,26]. Histone deficiency can also induce slowing of the replication fork although this appears to occur only after a long time, since transient histone depletion does not induce stress [27,28]. RPA consumption due to a large number of stalled forks can create an additional level of stress as the remaining stalled forks will collapse due to lack of RPA [29]. In addition, it was shown in yeast that reduced expression of replisome proteins can lead to replication stress and DNA damage [30]. Depletion of resources can occur as a result of extensive use due to over replication or due to a lack of coordination between pathways responsible for supplying such factors [31]. For example, early entry into S phase may cause the cell to begin replication before it has acquired the necessary factors [31].

Transcription interference can also induce replication stress. The replication timing program maintains coordination between replication and transcription in order to minimize interference between the two. Should this system be perturbed, collisions of the DNA polymerase and RNA polymerase can occur. During collision, the excess of the transcription and replication machineries creates topological constraint and supercoiling of the DNA, which can lead to knotting and prevent fork progression [32]. Transcription also results in the formation of R loops, which are ribonucleic acid (RNA):DNA hybrids that occur during transcription when the newly synthesized RNA anneals to the DNA leaving a displaced ssDNA strand. In the event of an R loop formation, the fork can be blocked by the RNA polymerase, the trinucleotide structure, or a lesion created in the ssDNA [33]. Transcriptional interference can be resolved by topoisomerase, helicase or ribonuclease H (RNase H), which prevents formation of R loops [34,35,36] and reduces topological stress.

Extent of origin usage is another critical factor, which can contribute to replication stress (reviewed in [37]). Intact origin usage has been implicated in a number of diseases such as Meier–Gorlin syndrome [38]. Re-replication of origins can occur when licensed origins initiate replication for a second time before mitosis, ultimately resulting in replication stress [39]. Over activation of origins can lead to stress and instability through depletion of nucleotides [40], RPA [29], or other replisome machinery or through disruption of the coordination between transcription and replication resulting in an increase in collisions [41], as described above. Conversely, under usage of origins has also been implicated in the accumulation of replication stress. Experiments in yeast [42,43], mice [44] and human cells [45] exhibiting licensing deficiency have demonstrated the link between origin sparsity and genomic instability. It has been shown that sufficient licensing of origins is needed to deal with replication stress [46,47], because the activation of dormant origins adjacent to stalled forks is a crucial mechanism for overcoming stress [48]. Thus, insufficient licensing may result in a lack of dormant origins and facilitate entry to mitosis before replication is completed [42,43,49].

The oncogene-induced replication stress model proposes that oncogenes facilitate replication stress [7]. Various well-established oncogenes have been shown to be involved in all the above described causes of replication stress (Table 1). Oncogene overexpression impairs normal cell cycle regulation and stimulates increased growth, abnormal replication initiation and abnormal transcription, all of which result in sustained proliferation and increased stress via the mechanisms described above. Increased cell metabolism associated with high levels of cell growth can contribute to ROS levels. Hyper-replication associated with cancer can lead to depletion of resources as well as early entry into S phase. Abnormal regulation of cell cycle processes can result in discoordination of transcription and replication resulting in increased collisions. Further, cancer has been associated with deregulation of origin usage through impairment of licensing mechanisms or due to general cell cycle deregulation. The mechanisms through which oncogenes induce replication stress are not exclusive as different oncogenes can induce stress through similar mechanisms. Additionally, a given oncogene can lead to stress through a number of different mechanisms occurring simultaneously. For example, a recent review of the contribution of *c-myc* to replication stress [50] suggested two coexisting mechanisms for fork stalling induction: over usage of origins resulting in depletion of resources and increased interference between transcription and replication.

### 2.2. Resolution of Replication Stress

There are a number of cellular mechanisms which are involved in the response to and resolution of replication stress. The DNA damage response (DDR) pathways respond to stress and DNA damage through either repair or cell cycle arrest. The main DDR pathway in the response to replication stress is the ATR pathway. RPA coats the ssDNA near a stalled fork and recruits and activates ATR. ATR localizes to and stabilizes the stalled forks [68,69] until either the source of stress is removed [20], or the region is replicated due to activation of nearby dormant origins [48,70,71] or through translesion synthesis [72]. The ATR pathway inhibits cell cycle progression by the activation of the intra-S checkpoint by activation of the checkpoint kinase 1 (Chk1). Phosphorylated Chk1 also inhibits further initiation of origins of replication and thus ensures more resources are available to deal with the current stress [29,73,74]. If the damage is insurmountable, cells will not return to the cell cycle but will rather enter senescence or apoptosis [75,76,77]. In this way, the DNA repair proteins together with the cell cycle arrest proteins protect the cell from reaching a state of genomic instability [75,78].

While the ATR pathway is activated by ssDNA, the ataxia-telangiectasia mutated (ATM) pathway is activated in response to DSBs and therefore also plays a role in the response to stress, particularly at collapsed replication forks associated with DSBs [79]. The Mre11-Rad50-Nbs1 (MRN) complex senses DSBs and recruits and activates ATM. ATM and the MRN complex can then facilitate homologous recombination to repair the DSB [80]. Further, localized ATM phosphorylates a number of substrates including checkpoint kinase 2 (Chk2) and H2A Histone Family Member X (H2AX). Phosphorylated H2AX, γH2AX, helps to recruit more ATM to the site and potentially opens the chromatin to allow for the recruitment of more proteins to fix the DSB [80]. The phosphorylated Chk2 acts as a checkpoint leading to cell cycle arrest. ATM also aids in fork restart through its interaction with DNA helicases involved in this process [81]. If the damage is not repaired, the ATM pathway will result in high levels of phosphorylated p53 which can lead to senescence or apoptosis [82].

The DNA damage tolerance (DDT) pathway, also known as post-replicative repair, is another pathway involved in the repair of stalled forks (reviewed in [5]). The DDT pathway is activated by PCNA ubiquitination which is induced by ssDNA at stalled forks [83]. DDT entails activation of specialized low fidelity translesion DNA polymerases which can bypass lesions in the DNA [72,84]. There are a range of different translesion polymerases, suitable for different types of damage and with different levels of fidelity (reviewed in [85]). In addition to translesion polymerases, DDT also employs template switching which is generally error-free and uses the already copied sister chromatid as a template to repair damage [86].

### 2.3. Replication Stress Leads to Genomic Instability, Common Fragile Sites Breakage, and Cancer

Failure to resolve replication stress can result in point mutations, loss of heterozygosity (LOH), insertions, deletions, translocations and aneuploidy [6,19,87]. Point mutations may stem from usage of translesion synthesis polymerases [84] whereas copy number variations commonly stem from aberrant restoration of stalled forks either by non-homologous end joining or through inaccurate fork restarting caused by error-free post-replication repair [88]. Mitotic catastrophe can occur in the event that the stalled forks are not resolved and the cell enters mitosis with only partially replicated DNA [6,21]. This can result in nondisjunction, anaphase bridges and lagging chromosomes leading to large-scale chromosomal damage [89,90,91,92].

While replication stress results in global instability, large genomic regions spanning a few 100 kilobases, termed common fragile sites (CFSs), are particularly vulnerable to replication stress and are more susceptible to double strand breaks (reviewed in [93]). Though these regions are typically stable under normal conditions, they frequently break under stress. The predisposition of CFSs to DNA damage has made fragile sites hotspots for translocations and deletions [94,95]. Many CFS properties contribute to their predisposition to DNA damage including increased transcriptional interference, late replication, origin shortage, AT-rich repeats and unique chromatin modifications [93]. Though CFSs are often associated with late replication, recently a new class of CFSs have been discovered, which are associated with early replication [96]. These early replication fragile sites (ERFSs) differ from CFSs (reviewed in [97]) and likely involve a different mechanism leading to their fragility. Late replicating CFSs are likely unable to complete replication before mitosis in replication stress conditions due to a combination of the aforementioned reasons [98,99], whereas ERFSs are probably mainly impacted by replication–transcription collisions [96].

On a broader scale, the oncogene induced replication stress model proposes that replication stress plays an important role in cancer progression. Studies have found increased levels of replication stress particularly in the early stages of cancers [58,60,100] suggesting that replication stress and the resulting genomic instability are primary hallmarks of cancer and not just downstream events [7]. Indeed, oncogene induced replication stress was shown to induce allelic imbalance at CFSs at the early stages of cancer even before the DDR was compromised [62,100]. Genome-wide studies have confirmed these findings and demonstrated that stress induced instability at CFSs is an early phenomenon in cancer development [101].

As cancer is associated with increased levels of replication stress [7], in pre-cancerous and cancerous cells CFSs are associated with deletions, translocations and amplifications [95,102]. The fragile site landscape in cancer is dependent on the unique combination of oncogenes in the cell [61]. Further support for the involvement of replication stress in generating genome instability in early stage of cancer, comes from mutation pattern analyses [31]. In cancer there are two main classes of mutations: driver mutations, referring to mutations which are selected for due to their conferring an advantage, and passenger mutations, referring to the many other mutations in the genome which seem to provide no benefit to the cancer cell. As passenger mutations do not supply any advantage to the cell and are therefore not influenced by selection, their pattern tells of the underlying mechanism of their generation during cancer development. Analyses of the pattern of the most common passenger copy number variations (CNVs) in many types of cancer revealed that they are enriched in late replicating regions or in very large genes. Similarly, it was demonstrated that the most common passenger point mutations are CG to TG located in large genes. While there are many mechanisms that can lead to CNVs in late replicating regions (see below), the enrichment of both CNVs and point mutations in large genes suggests that replication stress is a leading cause of these mutations and an early driver of cancer transformation.

### 2.4. The DDR in Cancer Progression and Therapy

Genomic instability induced by replication stress is not sufficient for generating complete cancer transformation. Under normal conditions, the cellular DDR drives defective cells into senescence or apoptosis and prevents transformation. Thus, cancer will develop only if oncogene induced replication stress is followed by evasion of the DDR. Accordingly, in preneoplastic cells, hyper-replication [60] and ROS [103] induced by oncogenic stress activate the DDR and ultimately lead to senescence, whereas the malfunctioning DDR of cancerous cells enables circumvention of cell death and ultimately results in complete transformation.

There is selective pressure for the mutation or elimination of tumor suppressors and other agents of the DDR pathway, such as downstream targets of ATR including p53, in order to allow the cell to proliferate malignantly. Studies have found that tissues in the early stages of cancer showed signs of functional apoptosis or senescence while only more advanced cancerous tissues were lacking in p53 and other DNA repair genes [7,58,100]. Similarly, Ras induction results in tumors only after evasion of the senescence checkpoints. Though high levels of Ras generally induce senescence, elimination of p53-dependent senescence checkpoints results in cancer transformation [104].

Though impairment of the DDR facilitates the progression of cancer, by conferring the ability to proliferate despite sustained damage, the DDR may also act as a powerful potential therapeutic target. Extreme DNA damage or impairment of DDR activity can ultimately result in mitotic catastrophe and cell death. This is due to the delicate balance of DDR and genomic instability in cancer cells. Mild levels of replication stress foster genomic instability and tumorigenesis, but high levels of stress will induce mitotic catastrophe [105]. Radiation and chemotherapies work according to this principle, as they induce general high levels of DNA damage which the cell cannot tolerate due to its defective DDR.

More recently, specific therapies have relied on increasing replication stress by directly targeting DDR pathways involved in the resolution of stress. Inhibitors of the DDR are useful targets in cancer therapy as their inhibition will speed up mitotic catastrophe (reviewed in [106,107]). Several Chk1 and ATR inhibitors are in clinical trials for anticancer therapy. Targeted therapies also aim to manipulate cell cycle regulator proteins such as WEE1 in order to speed up entry into mitosis before the DNA is fully replicated. Another major class of cancer drugs includes poly(ADP-ribose) polymerase 1 (PARP1) inhibitors that inhibit PARP1 proteins, which are crucial to repairing single strand breaks in DNA. Inhibition of PARP1 leads to accumulation of double strand breaks and ultimately cell death. In normal cells, fully functioning DDR as well as lower levels of cell growth, result in higher tolerance to the loss of these mechanisms and therefore these therapies are highly specific to cancer cells. Another potential target is gain of function p53 (GOF p53), a mutant p53 with oncogenic qualities. GOF p53 increases expression of necessary replication proteins and has recently been shown to stabilize replication forks preventing unmanageable levels of stress [108].

## 3. Replication Timing

### 3.1. Background

**Figure 1:**
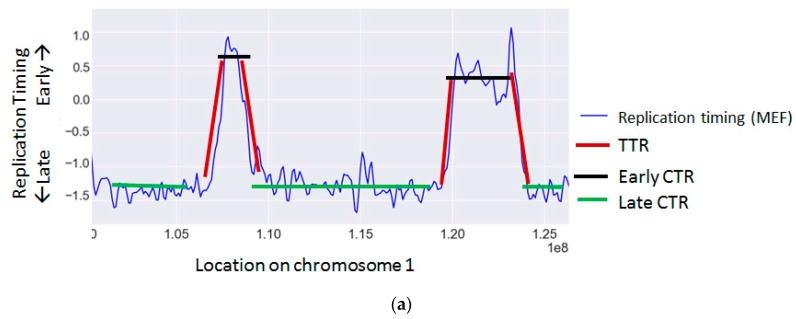

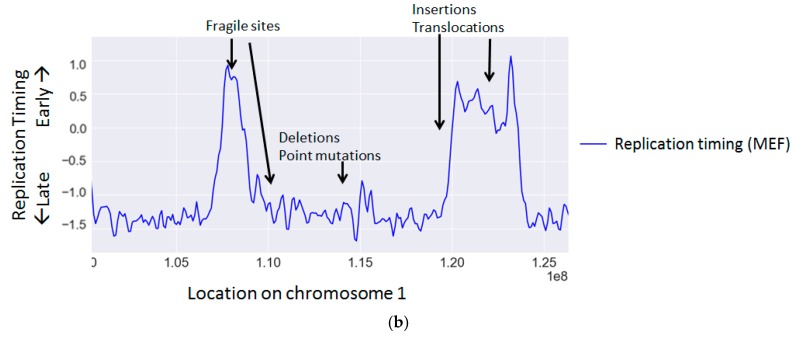
Representative replication timing profiles. (**a**) An example of a replication timing map of mouse embryonic fibroblasts (MEF) [111] for a 2 Mb-sized region labeled by region type: TTR (timing transition region) or CTR (constant timing region); (**b**) The same replication timing map with annotation of the regions prone to various types of mutations.

The cellular replication program is highly regulated and temporally coordinated according to a strict program [109]. During S phase of the cell cycle, each genomic region is replicated at a distinct time through the activation of an origin of replication. The time each region is replicated is a function of its distance from an active origin and of the time the origin was activated. Adjacent origins are usually activated simultaneously and give rise to large chromosomal regions that are replicated synchronously, called constant timing regions (CTRs). These regions are activated at various time points along S, giving rise to early, middle and late time zones. Between early and late zones there are large regions, in which the replication timing changes gradually, termed timing transition regions (TTRs) [9,110] (Figure 1a).

Replication timing has proven to be a measurable and stable epigenetic feature. In both humans and mice, it has been demonstrated that a large portion of the genome has a relatively constant replication timing, which does not vary between different developmental states and tissue types. However, the rest of the genome has been termed developmentally switching [112,113] and differs between specific tissue types.

The replication timing of a region, seems to reflect higher order genomic organization, since it correlates with basic genetic features such as the regional GC content, Giemsa banding, and gene density, as well as other epigenetic features such as chromatin state, and the three-dimensional (3D) structure of the genome [9,114]. Detailed analysis of the replication timing of individual genes has revealed a striking correlation between transcription and early replication. Expressed genes, such as constitutively transcribed housekeeping genes, replicate at early stages of S phase, whereas repressed tissue specific genes may replicate in most tissues at late stages of S phase and become early-replicating only in the expressing tissue [115].

There are two main methods used to determine replication timing. The first is based on copy number [111] and relies on the fact that in unsynchronized culture, the percentage of cells in S phase in which a locus has replicated is indicative of its replication timing. The second method is based on bromodeoxyuridine (BrdU) incorporation [116]. Cells are exposed to BrdU and sorted to early and late fractions in S phase. BrdU labeled DNA is immunoprecipitated from the different S fractions and measured in order to determine the replication timing.

### 3.2. Effects of Replication Timing on Mutation Rates and Structural Variations

Replication timing is known to correlate with the rate of many types of mutations and chromosomal aberrations (Table 2 and Figure 1b) (reviewed in [117]).

Point mutation rates are associated with replication timing in a range of organisms. Studies of divergence between chimpanzee and human [118,119], rat and mouse [119,122], and within different *Drosophila* species [123], have demonstrated increased mutation prevalence in late replicating regions. Analyses of single nucleotide polymorphism (SNP) data in both human [118] and mouse [119] have confirmed this bias. This bias has even been demonstrated in yeast, where the *URA3* gene, encoding orotidine-5′-phosphate decarboxylase, was inserted in different replication timing zones and displayed corresponding changes in mutation rate [131]. In addition to these studies in germline mutation rates, studies using multiple cancer types [124,125] as well as studies of individual cancers such as liver [126] and lung cancer [129], have also exhibited a bias for higher point mutation rates in late replicating zones. Despite the general association of mutation rate and replication timing, mutation signature analyses revealed that the correlation appears to be extremely nuanced and different mutation signatures correlate differently to replication timing [120,130,146].

Larger structural changes in DNA such as amplifications and deletions have also been linked to replication timing patterns. Studies in flies discovered enrichment of duplications in late regions and deletions in early regions [136,137]. However, studies in human cancers have found the opposite trend demonstrating that amplifications are more likely to be in early regions whereas deletions tend to be in late regions [133]. It is unclear whether this difference is due to the difference in species or due to the cancerous effects. As noted earlier, most common fragile sites are enriched in late replicating regions. The fact that replication timing varies between tissues has been used to explain the variation in CFS instability between different tissue types, supporting the strong connection between replication timing and fragility [142]. Certain late replicating regions do not manage to complete replication by the end of S phase leading to fragility [99,143,144]. Other CFSs, which generally replicate in early/mid S may be delayed to late S replication due to stress and are thereby made fragile [147,148,149].

Several studies in cancer cells found that rearrangements or translocations occur preferentially in early replicating regions [139,140]. However, one study found a subset of rearrangements in specific cancers, which occur in particularly late regions [138], suggesting that this phenomenon may be cancer type specific. Interestingly, translocation partners tend to be from the same timing regions [141], probably reflecting the spatial proximity of regions replicating at the same time [150,151].

TTRs also exhibit enrichment of specific mutation types. As TTRs are regions located between early and late zones, which contain few or no origins [109,152,153,154], each replication fork replicates a large genomic region and is therefore more prone to fork stalling and collapse [155]. Indeed TTRs exhibit higher levels of SNP density [121] as well as amplifications [134].

The mechanism, by which replication timing may affect mutation rates, appears to be multifaceted and a number of theories have been proposed. It has been shown that the increased mutation rate characteristic of late replicating regions impacts different mutation types similarly, suggesting that the bias stems from the general accumulation of ssDNA in late regions [118]. However, other more recent studies, have found that the mutational bias differentially favors specific mutation types [120,146,156] and suggests that factors unique to the different stages of S phase may be contributing to the differential rates.

It has been suggested that the differential mutation rates along S phase are formed due to different replication and repair machinery present at different stages of S. For example, a study in yeast demonstrated that elimination of the rev1 translesion polymerase, which is more active in late S, removed the bias of point mutations in late regions [131]. Studies in humans have also pinned mutational correlation on differential DNA machinery, for example by showing that mutations are not enriched in late regions in mismatch repair deficient cells [127]. Similarly, the replication timing bias of CNVs can be explained by their method of formation. It has been shown that homologous recombination based CNVs are enriched in early replicating regions whereas CNVs generated by other mechanisms such as non-homologous end-joining (NHEJ) and microhomology-mediated break-induced replication occur more frequently in late replicating regions [120]. Another potential mechanism contributing to the replication timing mutational bias is the temporal variation in the composition of the dNTP pool, which favors different nucleotides at different times in S phase [156]. Alternatively, the connection between replication timing and mutational frequency may be impacted by other factors, which correlate closely with replication timing such as open versus closed chromatin state which may affect the accessibility of the DNA to different DNA repair mechanisms [157].

### 3.3. Replication Timing Changes and Cancer

A number of replication timing changes have been linked to cancerous transformation (Table 3). In a genome-wide study of replication timing in cancer [10], 17 pediatric leukemia tumors were analyzed to determine the changes in replication timing after cancer transformation. The cancerous replication timing profiles varied in comparison to normal B and T cell replication timing. Additionally, replication timing heterogeneity was found within the different tumor specimens. Interestingly, replication timing changes common to all cancer specimens included the region next to the Runt-related transcription factor 1 (*RUNX1*) locus, a common leukemic translocation site though only some specimens displayed the actual translocation itself. This suggests the possibility that the replication timing change is an early step predisposing the cancer cell to the translocation. Another study in tumor-derived cell lines [158] also found a connection between translocations and replication timing, however, suggesting that rearrangements can result in chromosome-wide replication delays.

Studies in different cancer cells have reported changes in replication timing of specific cancer-related genes. Among these are tumor suppressor genes like tumor protein P53 (*p53*), retinoblastoma 1 (*RB1)*; DNA repair related genes like *ATM*; and classical oncogenes like *c-myc*, *HER-2/neu* [11,159,160]. These replication timing changes can be specific to one allele resulting in asynchronous replication of the two alleles [11,159,160,162], or biallelic, causing a uniform change of the replication timing of the gene [11]. The B-cell lymphoma 2 (Bcl2) gene, which is frequently translocated in human follicular lymphoma is associated with a change from middle to early replication in the translocated gene in correlation with its overexpression [163].

### 3.4. Causes of Replication Timing Changes in Cancer

It is unclear what exactly leads to the changes in replication timing associated with cancer. It has been shown in yeast that most global changes in replication timing stem from local changes in replication fork speed and origin activation [164]. Thus, oncogene-induced replication stress, which slows down fork progression and inhibits global initiation of origins of replication, may play a role in changing the replication timing landscape. Indeed, specific oncogenes have been shown to modulate the replication program. For example, the *c-myc* proto-oncogene is involved in replication initiation and its deregulation can result in premature origin firing [53] potentially bringing about global changes in the replication timing program.

Misregulation of other replication-related genes can also effect changes in the replication timing program and have been implicated in the development of cancer [165]. In yeast, forkhead box proteins [166] as well as the replication timing regulatory factor 1 (Rif1) protein [167] play important roles in the maintenance of accurate replication timing [166]. The role of Rif1 in replication timing has been demonstrated in mice [168,169] and humans [170] as well. Changes in the expression of proteins involved in the minichromosome maintenance (MCM) complex, which is involved in initiation of origins of replication, may also affect replication timing since the amount of MCM complexes at an origin influences its timing [171]. Interestingly, polymerase theta also plays an important role in determination of replication timing, and its depletion or overexpression leads to large scale replication timing changes [172].

In addition to changes in the expression of specific genes associated with DNA replication, replication timing may be indirectly affected by alterations to closely related epigenomic features. For example, changes in histone modifying proteins can lead to changes in the chromatin state, which may then alter the replication timing program [173,174].

Translocations can affect replication timing locally since the edges of the translocated gene may propagate its timing to its new neighbors [141]. Alternatively, translocated genes may adopt the timing of their new region [163]. Translocations may also influence replication timing on a more global level by delaying replication of the entire chromosome [158].

### 3.5. Effects of Changes in Replication Timing in Cancer

Replication timing changes can result in genomic instability and cancer development in a number of ways. First, replication timing changes may potentially influence the progression of cancer through their impact on the gene expression profile. The causal relationship between replication timing and expression is quite complicated. Though replication timing is associated with gene expression [110,175] this correlation does not hold true for all genes [112]. Further, it is unclear if replication timing changes are sufficient to induce changes in expression [176]. Nonetheless, changes in the replication timing of specific genes in cancer cells have been associated with their overexpression [11], possibly due to their repackaging as open chromatin [177]. It is possible that genes which are switched to earlier replication may exhibit higher expression levels, though further research is required to elucidate this point.

Second, replication timing can affect cancer development through its mutational impact. As established earlier, replication timing plays an important role in determining the mutational landscape of the genome. In fact, it has been shown that cell type specific replication timing can best explain the mutational landscape of different cancer tissues [127,178]. This suggests that replication timing may be a contributing factor in cancer progression as changes in replication timing of important oncogenes or tumor suppressor genes may lead to their further mutagenesis. Indeed, it has been suggested that changes in the replication timing of the *RUNX1* locus, precede chromosomal breakage and translocations common in leukemia [10].

Third, particularly aberrant changes in the replication timing program can induce stress and thus propagate genomic instability. Unscheduled origin licensing and discoordination of replication forks can impede on fork progression and overload the system resulting in a lack of resources in the form of nucleotides or other factors [6]. A change in replication timing may also interfere with the proper coordination of replication and transcription resulting in increased collisions [41]. Delayed replication induced by cancer-related chromosomal rearrangements has also been associated with overall instability and increased acquisition of translocations [158].

## 4. Conclusions

Faithfulness of the DNA replication program is crucial to cell viability and its impairment can ultimately lead to the development of cancer. Both management and prevention of replication stress as well as maintenance of ordered replication timing are crucial to cell viability and the prevention of genomic instability. Further studies are required in order to comprehensively address the relationship between replication timing and replication stress. Insight into their interplay may shed light on the development of genomic instability during aberrant DNA replication.

Increased replication stress is a hallmark of cancer progression and can be particularly useful as both a biomarker and as a therapeutic target. Currently, biomarkers of stress rely mainly on the appearance of γH2AX. Discovery of more biomarkers, specifically those also involved in the ATR pathway, may be particularly effective as a diagnostic method for detecting levels of replication stress in early stages of cancer development. Further understanding of replication stress mechanisms and DDR pathways could aid in the discovery of additional therapeutic targets.

Despite many years of research, there are limited new findings highlighting the association between replication timing and cancer. As sequencing costs decrease and experimental methods improve we expect to gain new insights into replication timing and its connection to genomic instability. Replication timing data from cancer tissues themselves will not only improve our grasp of the impact of specific replication timing changes, but may also serve as an important step in the establishment of replication timing as a biomarker in cancer diagnostics [10]. Furthermore, time course studies of replication timing changes in cancer progression will advance our understanding of the specific causes and effects of such changes.

The limiting factor in such research is the availability of large amounts of dividing cancer cells. Recently, new methods have been developed which will aid in the study of replication timing in human cancers. For example, it was demonstrated that mouse xenografts retains the replication timing of the original human transformed cells in acute lymphoblastic leukemia (ALL) cancers [179]. An alternative approach would require measuring replication timing from few thousands of cells. This advancement would open the field for in vivo studies and will allow genome-wide study of the replication timing in cancer cells.

Recent studies [180] have expanded analyses of the replication program to include data on processes differentially affecting the leading and lagging strands. These studies remain to be expanded and will likely shed light on more mutational processes involved in replication, which contribute to genomic instability.

## Figures and Tables

**Table 1:** Oncogenes involved in replication stress.

Gene	Mechanisms	References
*c-myc*	Accelerated S phase, increased origin activity, ROS, transcriptional interference	[51,52,53,54,55,56,57,58]
*Cdt1*	Re-replication	[59]
*H-RasV12*	Oxidative stress, hyperreplication	[55,58,60,61]
*E2F1*	Deregulated replication , Oxidative stress	[57,62,63]
*MOS*	Unknown	[58]
*Cdc6*	Unknown	[58]
*Cyclin E*	Deregulated replication, deficient licensing, transcriptional interference, origin over usage, nucleotide depletion	[26,58,61,62,64,65,66]
*Cdc25A*	Deregulated replication	[62,67]
*HPV-16 E6/E7*	Nucleotide depletion	[26]

*c-myc*: MYC proto-oncogene; *Cdt1*: Chromatin Licensing And DNA Replication Factor 1; *H-RasV12*: HRas proto-oncogene mutant (constitutively active); *E2F1*: E2F Transcription Factor 1; *MOS*: MOS Proto-Oncogene, Serine/Threonine Kinase; *Cdc6*: cell division cycle 6; *Cdc25A*: Cell Division Cycle 25A; *HPV-16 E6/E7*: Human papillomavirus 16 E6/E7 ; ROS: Reactive oxygen species.

**Table 2:** Studies linking replication timing to mutations.

Mutation type	Measurement	Higher in	References
Germline point mutations	Human SNP	Late and TTR	[118,119,120,121]
	Mouse SNP	Late	[119]
	Mouse–rat divergence	Late	[119,122]
	Human–chimp divergence	Late	[118,119]
	*Drosophila* divergence	Late	[123]
Somatic point mutations	Human cancer	Late	[124,125,126,127,128,129,130]
Yeast point mutations	Yeast *URA3* gene	Late	[131,132]
Insertions	Human cancer	Early and TTR	[126,133,134]
	Human iPSC	Early	[135]
	Fly	Late	[136,137]
Translocations	Human cancer	Early (and late in [138])	[126,133,138,139,140]
	Mammalian divergence	TTR/early	[134,141]
Deletions	Human cancer	Late	[126]
	Human iPSC	Late	[135]
	Fly	Early	[136]
Fragile sites	Cancer	Early or late	[96,99,142,143,144]
LOH	Cancer	Early	[145]

SNP: single nucleotide polymorphism; TTR: timing transition regions; *URA3*: yeast gene encoding orotidine-5′-phosphate decarboxylase; iPSC: induced pluripotent stem cells; LOH: loss of heterozygosity.

**Table 3:** Studies linking replication timing changes to cancer.

Replication Timing Change	Genes	Cancer Type	Reference
Global replication timing changes	*Global changes*	Bone marrow from ALL patients	[10]
Asynchronous replication	*HER-2*, *AML1*, *RB1*, *c-myc*, *p53*	Peripheral blood lymphocyte of cancer patients, MCF10CA1a	[11,159,160,161,162]
Replication timing changes of cancer-related genes	*p53*, *ATM*, *c-myc*, *RAD51*, *PTEN*, *translocated Bcl2*	MCF10CA1a, SU-DHL-6, Jurkat	[11,163]

*HER2*: human epidermal growth factor receptor 2; *AML1*: acute myeloid leukemia 1 protein; *RB1*: Retinoblastoma 1; *c-myc*: MYC proto-oncogene; *ATM*: ataxia-telangiectasia mutated; *Bcl2*: B-cell lymphoma 2; ALL: Acute lymphoblastic leukemia; MCF10CA1a: malignant human breast cell line; SU-DHL-6: Stanford University-Diffuse Histiocytic Lymphoma-6 cell line.

## Abbreviations

DNA	Deoxyribonucleic acid
S phase	Synthesis phase
ssDNA	Single stranded DNA
DSB	Double strand breaks
RPA	Replication protein A
ATR	Ataxia telangiectasia and Rad3-related protein
ROS	Reactive oxygen species
dNTP	Deoxynucleotide triphosphate
RNA	Ribonucleic acid
RNase H	Ribonuclease H
DDR	DNA damage response
Chk1	Checkpoint kinase 1
MRN	Mre11-Rad50-Nbs1
Chk2	Checkpoint kinase 2
H2AX	H2A histone family member X
DDT	DNA damage tolerance
LOH	Loss of heterozygosity
CFS	Common fragile site
ERFS	Early replication fragile site
CNV	Copy number variations
PARP1	Poly(ADP-ribose) polymerase 1
GOF p53	Gain of function p53
CTR	Constant timing regions
TTR	Timing transition regions
MEF	Mouse embryonic fibroblasts
BrdU	Bromodeoxyuridine
SNP	Single nucleotide polymorphism
NHEJ	Non-homologous end-joining
RUNX1	Runt-related transcription factor 1
p53	Tumor protein P53
RB1	Retinoblastoma 1
Bcl2	B-cell lymphoma 2
Rif1	Replication timing regulatory factor 1
MCM	Minichromosome maintenance
ALL	Acute lymphoblastic leukemia
